# Effect of Frying Conditions on Self-Heating Fried Spanish Mackerel Quality Attributes and Flavor Characteristics

**DOI:** 10.3390/foods10010098

**Published:** 2021-01-05

**Authors:** Lili Chang, Songyi Lin, Bowen Zou, Xiaohan Zheng, Simin Zhang, Yue Tang

**Affiliations:** 1 School of Food Science and Technology, Dalian Polytechnic University, Dalian 116034, Liaoning, China; changlili0818@163.com (L.C.); linsongyi730@163.com (S.L.); Zbw790441205@163.com (B.Z.); XH1075217650@163.com (X.Z.); zhangsm0701@163.com (S.Z.); 2 National Engineering Research Center of Seafood, Dalian 116034, Liaoning, China

**Keywords:** Spanish mackerel, food quality, headspace-gas chromatography-ion mobility spectrometry (HS-GC-IMS), volatile organic compounds (VOCs)

## Abstract

In this study, we investigated the effects of different frying conditions on the quality characteristics of fried Spanish mackerel (*Scaberulous niphonius*) to address the food quality degradation of self-heating fish products after frying, sterilization, and reheating. Furthermore, the effect of different moisture contents (65%, 60%, 55%, and 50%) of fried Spanish mackerel on texture, color, and microstructure after sterilization and self-heating were examined. The flavor fingerprints of different frying temperatures (140 °C, 160 °C, 180 °C, and 200 °C) coupled with the optimal moisture content were identified; furthermore, volatile organic compounds (VOCs) were studied using headspace-gas chromatography-ion mobility spectrometry (HS-GC-IMS) with principal component analysis (PCA). The results indicated that the shear force value significantly increased, while the hardness and chewiness significantly decreased simultaneously with decreasing moisture content. Samples containing 65% moisture content showed the highest L*, a*, and W values, while their b* value was the lowest, and the most clearly visible fibrous veins with tiny cracks could be observed in them. Samples fried at 160 °C and 65% moisture content exhibited the richest VOCs, with a greasy or fried aroma. Based on the PCA, there were significant differences in the sample VOCs under different frying conditions. In summary, among all treatments, frying at 160 °C with 65% moisture content resulted in the highest food quality of fish filets. The results of this study could provide a theoretical basis for improving the food quality of self-heated fish products.

## 1. Introduction

Consumers have always favored seafood products due to their tenderness, unique flavor, and delicious taste. Because they have abundant nutrients, including essential amino acids, proteins, lipids, and minerals, aquatic products are deemed vital nutrient sources highly beneficial for human health. Currently, aquatic products are mainly present in the market in the form of dried products, pickles, and canned food (hard or soft packed). In the past years, self-heating foods have become increasingly favored by consumers who like to eat warm meals but do not have enough time to cook for themselves. Self-heating food, a type of food heated with a flameless self-heater, is mainly heated by an exothermic reaction agent set in the package and can be quickly reheated. Due to thermal sterilization under high temperature and high pressure, self-heating food safety is guaranteed, but food color, flavor, and nutritive values deteriorate during the self-heating process. The current research on self-heating food focuses on packaging [1] and shelf-life prediction [2,3]. However, the possibilities of improving the nutritive quality of self-heating aquatic products during processing have not been fully explored yet.

The cooking process can strongly impact the texture, color, and other significant quality factors of the final product [4], which are crucial consumer satisfaction-related attributes. During heat processing, collagen disintegration or protein denaturation can cause the partial or complete decomposition of the muscle fiber structure; moreover, the springiness and hardness were found to be decreased, in addition to other textural parameters [5]. Zeng et al. [6] observed that cooking treatment could induce serious shrinkage of muscle fibers of salted-dried fish, resulting in textural changes due to increased sample springiness, as well as reduced hardness and chewiness. During deep-frying, chicken breast filets showed surface muscle fiber splitting as soon as they were placed into the oil, and the depth of damage increased as time progressed, including a significant increase in shear force [7]. Additionally, color is a vital quality factor of fried food, influencing the choice of the consumers, who prefer to choose products with a light color; hence, the conditions of the frying process should be strictly controlled [8].

Flavor is a vital unique sensory characteristic that affects consumer likeability and acceptability of meat products [9]. Frying can contribute to numerous chemical reactions, causing the formation of volatile organic compounds (VOCs) and consequently delicious and unique flavors. The chemical reactions mainly include thermal decomposition of certain ingredients from raw materials, the Maillard reaction between reducing amino acids and sugar compounds, and the lipid degradation of fatty acids from the frying oil [10]. Headspace-gas chromatography-ion mobility spectrometry (HS-GC–IMS) is a specific and simple method for identifying VOCs. Recently, HS-GC–IMS has been proven to be a helpful approach for analyzing and recognizing VOCs with different characteristics in foods, including dry-cured fish [11], *Tricholoma matsutake* Singer [12], sea cucumber peptide powders [13], peppers [14], and boiled Wuding chicken [15].

Spanish mackerel (*Scaberulous niphonius*), one of the most abundant marine fish, is distributed in the East China Sea, Yellow Sea, and off Western Japan [16]. The demand for Spanish mackerel in markets of China, Japan, Korea, and other countries has increased notably over the past decade due to its tenderness, delicious taste, and nutritional value. In the present study, Spanish mackerel was used as a representative sample to identify the quality attributes and flavor characteristics of self-heating fried aquatic products during processing. This study aimed to investigate the changes in fish filet texture characteristics, color parameters, and microstructure with different moisture contents after frying, sterilization, and self-heating. The effect of different frying temperatures with optimal moisture content on the VOCs was measured. Flavor fingerprints were identified using HS-GC-IMS. The information reported in this study could be useful for producing self-heating aquatic food products that could improve flavor and food quality.

## 2. Materials and Methods

### 2.1. Materials and Chemicals

Frozen Spanish mackerel was obtained from a local market in Dalian, China, kept in a polyethylene bag, and stored at −20 °C until analysis. The fish were washed, and the viscera and bones were removed. The size of each fish used in this study was 2 × 2 × 2 cm (8.50 ± 0.5 g). A petroleum ether (analytical grade) was obtained from Kemiou Chemical Reagent Co., Ltd. (Tianjin, China). Soybean oil purchased from a local supermarket was chose according to the oils of the self-heating products of the famous food enterprises (Sanquan and Kaixiaozao Foods Co., Ltd. et al., Henan and Shanghai, China) in China.

### 2.2. Preparation of Fried Fish Filets

Spanish mackerel samples (six samples for each experimental group) were fried in a deep-frying pan (Guangzhou Aishqi Electrical Technology. Co., Ltd., Guangzhou, China). The frying step gave a good shape, attractive color, and flavor to fish filets and it was beneficial to maintain the fish filets’ quality during the subsequent processing. After frying, each group of fish sample was drained of excess oil for 15 min with oil-absorbing sheets and cooled to room temperature. The fried fish filets were then vacuum packed, sterilized at 105 °C for 10 min, 110 °C for 20 min, and 121 °C for 20 min using a backpressure high-temperature cooker (Guangzhou Biaoji Packaging Equipment Co., Ltd., Guangzhou, China). Before analysis, the samples were heated at 100 °C for 10 min to simulate the self-heating process, and this condition was provided by Sanquan Foods Co., Ltd. (Henan, China) which produces self-heating food in China.

### 2.3. Moisture Content Analysis

The samples were used for moisture content analysis according to the Association of Official Analytical Chemists [17], and curves showing variations in frying time (1–5 min) at different frying temperatures (140 °C, 160 °C, 180 °C, and 200 °C) were plotted to determine the moisture content of the samples.

### 2.4. Textural Analysis

After frying, sterilization, and reheating, the textural properties of the samples, which were cut into 1 × 1 × 1 cm sections, were determined using a texture analyzer (TA-XT2i; Stable Micro Systems, Surrey, UK). The texture profile analysis was performed according to the method of Dong et al. [18] with some modifications. The testing parameters were as follows: pre-test speed 2.0 mm/s, test speed 1 mm/s, post-test speed 2 mm/s; compressed depth 30%, time interval 5.0 s, compressed time 2, and model P50 head.

Shear force was measured using a rectangular probe (HDP/BS), as described by Dong et al. [18]. Each sample was subjected to pre-test speed, test speed, and post-test speed of 1, 1, and 10 mm/s, respectively. The distance was 35.0 mm.

### 2.5. Color Measurement

Color was determined instrumentally using an Ultra Scan PRO color photometer (Hunter Lab, Reston, VA, USA). A whiteboard was used as background for color measurement, and the test pattern was eyelet reflection [19]. Each sample was used to determine L* (lightness), a* (redness), and b* (yellowness) values. The formula for calculating the values of whiteness was as follows:

W = 100 − [(100 − L*)^2^ + a*^2^ + b*^2^]^1/2^

### 2.6. Scanning Electron Microscopy (SEM)

A scanning electron microscope (Model: JSM-7800F; JEOL, Tokyo, Japan) was used to determine the surface morphology of the samples (after frying, sterilization, and reheating). To obtain clear SEM images, the fried samples were treated by defatting with petroleum ether for 12 h before examination via SEM. The samples were attached to a bronze metal slide with double-sided adhesive tape and sputter-coated with gold prior, and they were observed at 50× magnification.

### 2.7. HS-GC–IMS Analysis

The VOCs of samples fried at different frying temperatures (140 °C, 160 °C, 180 °C, and 200 °C) with the optimal moisture content were measured according to the method of Li et al. [12] using a gas chromatography-ion mobility spectrometer (GC-IMS) (FlavorSpec^®^) from Gesellschaft für Analytische Sensorysteme mbH (G.A.S., Dortmund, Germany). Briefly, 2 g of the minced muscle was added into a headspace glass sampling vial and injected (500 μL) automatically using a heated syringe (85 °C) after 20 min of incubation at 60 °C. The conditions of the analysis were as follows: 2 mL/min for 2 min, 10 mL/min for 8 min, 100 mL/min for 10 min, and 150 mL/min for 10 min to stop. The drift gas flow was set to 150 mL/min. The retention index (RI) of the analytes was calculated using n-ketones C4–C9 as external references with chromatographic conditions similar to those of the samples. Two-dimensional fingerprint maps, topographic plots, and clustering of differential volatile organic compounds of samples under diverse frying conditions were established, with the results analyzed using the Laboratory Analytical Viewer, Reporter, Gallery Plot, Dynamic PCA, and GC × IMS Library Search database.

### 2.8. Statistical Analysis

All samples were analyzed independently, and all experiments were performed in triplicate. Values are expressed as the mean ± standard deviation (SD). The experimental data were analyzed and processed using the IBM SPSS statistical software package 22 (SPSS Inc., Chicago, IL, USA). The differences were analyzed for significance by analysis of variance (ANOVA). Duncan’s multiple range test of means (*p* < 0.05) was used to test for significant differences.

## 3. Results and Discussion

### 3.1. Fish Filet Moisture Content Determination under Different Conditions

**Figure 1:**
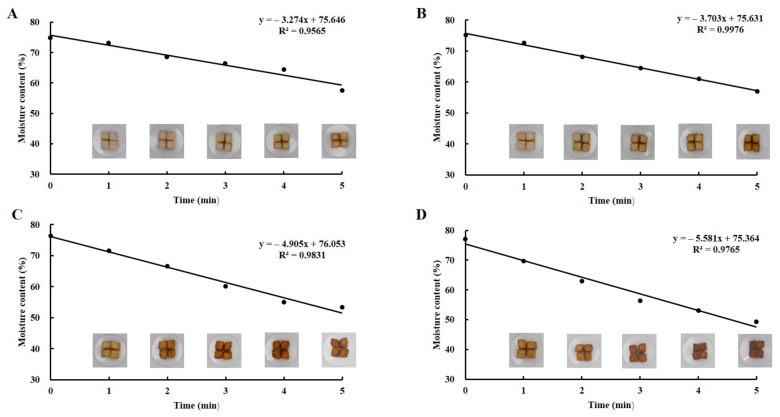
The curves of moisture content of fish filets and equation of fish filet moisture loss at different frying temperatures and times: 140 °C (**A**), 160 °C (**B**), 180 °C (**C**) and 200 °C (**D**).

The moisture content of fried fish filets has a significant influence on food quality. Different moisture contents result in different levels of fish tenderness. The moisture content curves of fish filets at different frying temperatures and times are shown in Figure 1. The results showed that the moisture content of fish filets decreased continuously with increasing frying time, and the higher the frying temperature, the more rapidly the moisture content of fish filets decreased. For raw filets, the moisture content was 75.87 ± 1.06%, which was in good agreement with our previous studies [20]. After the fish filets were fried at 140 °C, 160 °C, 180 °C, and 200 °C for 5 min, the moisture content decreased by 17.26%, 18.27%, 23.13%, and 27.29%, respectively. During frying, heat is transferred by convection from the hot oil onto the fish filet surface and then transmitted into the center, causing moisture content evaporation from the inside of the fish filet toward the outer layer [21]. This might be the cause of the decreasing moisture content. The samples with 65%, 60%, 55%, and 50% moisture contents were selected for further studies based on the moisture content curves to determine the optimal moisture content of self-heating fried fish filets.

### 3.2. Effect of Different Moisture Content on Self-Heating Fried Fish Filet Texture

**Figure 2:**
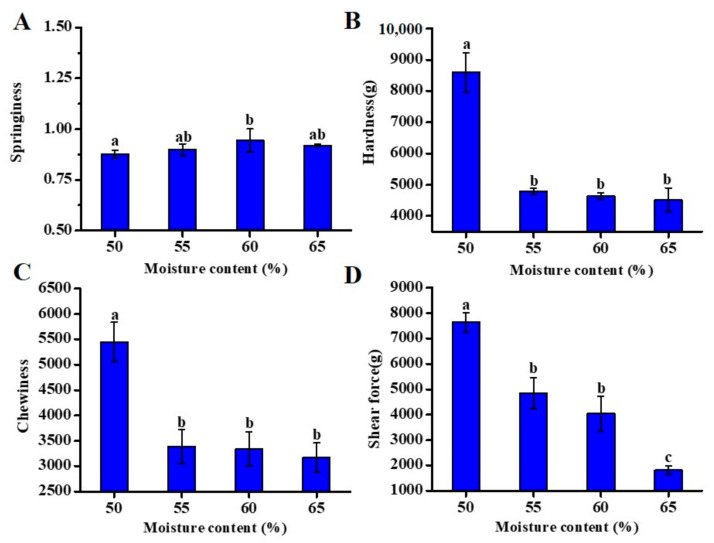
Effect of moisture content on the textural properties of fish filets: springiness (**A**), hardness (**B**), chewiness (**C**) and shear force (**D**). Different letters represent significant differences (*p* < 0.05).

Texture analysis was used to measure factors such as hardness, springiness, chewiness, and shear force. These factors are the main variables used to describe the characteristics of the tissue and are considered to be vitally significant factors affecting consumer satisfaction [22]. Figure 2A shows the moisture content-dependent change in the springiness of fish filets. There was no significant difference in springiness during the processing, in the case of any moisture content (*p* < 0.05). This illustrated that the different moisture contents had little effect on springiness. As the moisture content decreased, fish filet hardness (Figure 2B) significantly increased (*p* < 0.05). The hardness of fish filets with a moisture content of 65% was 4529.83 ± 373.75 g, increasing to 8593.71 ± 623.43 g when the moisture content of fried fish filets dropped to 50%. The loss of moisture strengthened the mechanical energy and viscosity, resulting in a compact structure in the case of fried food [23]. Similar to the hardness values, the sample chewiness values (Figure 2C) showed a significantly decreasing trend with a simultaneous increase in water content (*p <* 0.05), indicating that when the water content was 50%, sample taste worsened compared to the other three groups. The shear force value (Figure 2D) increased significantly during frying (*p <* 0.05). The lower the shear force value was, the more tender the fish became. Heat treatment caused molecular stretching and dissociation in the structure of the myofibrillar protein, and the exposed sulfhydryl groups were oxidized into more stable disulfide bonds. Disulfide bond polymerization makes the protein structure denser, and thus, myofibrillar protein condenses and contracts, causing muscle loss, which might be the reason for the increased shear force at high temperatures [24].

### 3.3. Effect of Different Moisture Content on Self-Heating Fried Fish Filet Color

**Figure 3:**
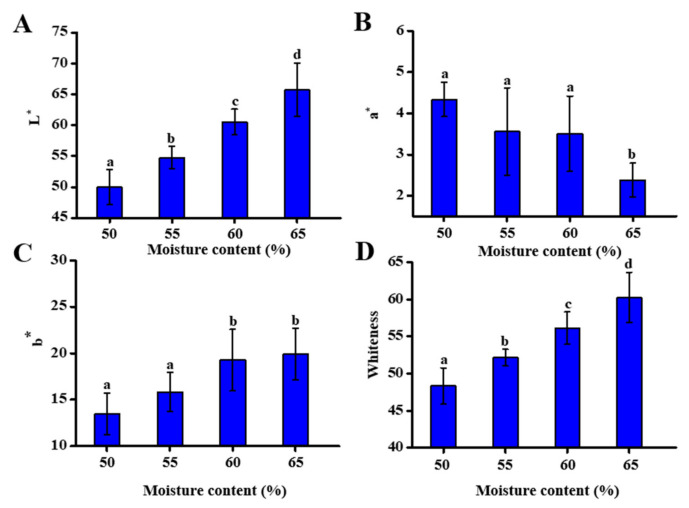
Effect of moisture content on the color attributes of fish filets: L* (**A**), a* (**B**), b* (**C**) and whiteness (**D**). Different letters represent significant differences (*p* < 0.05).

The color of aquatic products is an important quality control and consumer acceptance indicator [25]. The determination of fish filet color attributes (Figure 3) suggested that the changes in lightness (L*), redness (a*), yellowness (b*), and whiteness (W) all contributed to the overall color variations. L* values (Figure 3A) reduced progressively from 65.75 ± 4.30 to 49.98 ± 2.84, with a reduction in moisture content due to the loss of moisture and the weakening light refraction, suggesting that fish filets became darker during the heat treatment process. a* value magnitude (Figure 3B) significantly increased with the decreasing moisture content. When the moisture content of the fish filets was 50%, the a* value reached the highest value of 4.34 ± 0.42. Regarding the b* value (Figure 3C), fish filets with moisture contents of 65% and 60% were higher than those with 55% and 50% moisture content. The fish filet whiteness values decreased significantly (*p* < 0.05) with the decreasing moisture content (Figure 3D). These results indicated that as the moisture content decreased, the color of the fish progressively became darker. The reason for such darkening was that prolonged processing at higher temperatures was conducive to Maillard browning reactions, the caramelization of sugar, and the oxidization of the samples [26].

### 3.4. Effect of Different Moisture Content on Self-Heating Fried Fish Filet Microstructure

**Figure 4:**
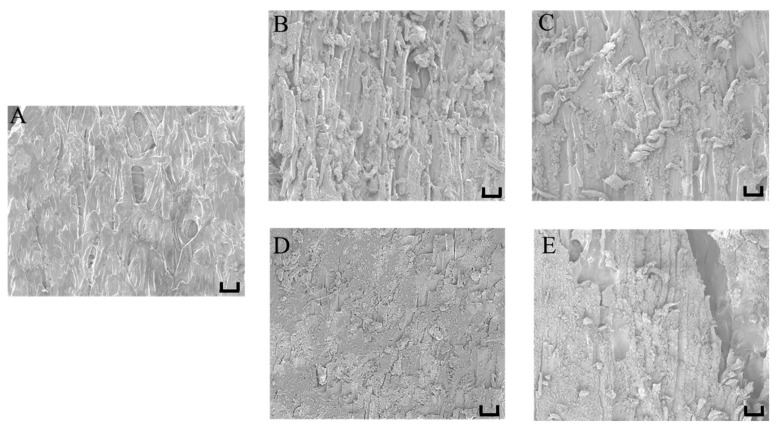
Effect of moisture content on the microstructure of fish filet samples by SEM images (50×). (**A**) fresh, the fish filets fried to (**B**) 65%, (**C**) 60%, (**D**) 55%, (**E**) 50% moisture content. Scale bar = 100 µm.

The microstructural changes in fish muscle could reflect the textural changes related to the association between water and protein molecules [27]. Similar to previous studies [28,29], we also observed great differences between raw and cooked fish muscles. As shown in the SEM images in Figure 4, the muscle fibers of the cooked samples were clearer than those of the raw samples. After the heat treatment, the muscle fibers showed slight fragmentation, and myofibril separation was also apparent. The fried fish filet with 65% moisture content exhibited clearly visible fibrous veins with tiny cracks, and its myofibrils were arranged neatly and tightly, implying a tender fish filet taste. As the moisture content decreased, the muscle fibers of the samples became denser with more compact fiber arrangements, especially the fried fish filets with 50% moisture content, resulting in gradually harder samples, which verified the conclusion of hardness in Figure 2B. During the heating process, collagen and myofibril degeneration, coupled with endomysium and perimysium decomposition, caused muscle fiber contraction and aggregation [30].

According to the analysis of the above-mentioned results, the fish filets with a moisture content of 65% exhibited the best quality and were selected for further investigations.

### 3.5. HS-GC–IMS Topographic Plots for Different Frying Temperatures of Self-Heating Fried Fish Filets

**Figure 5:**
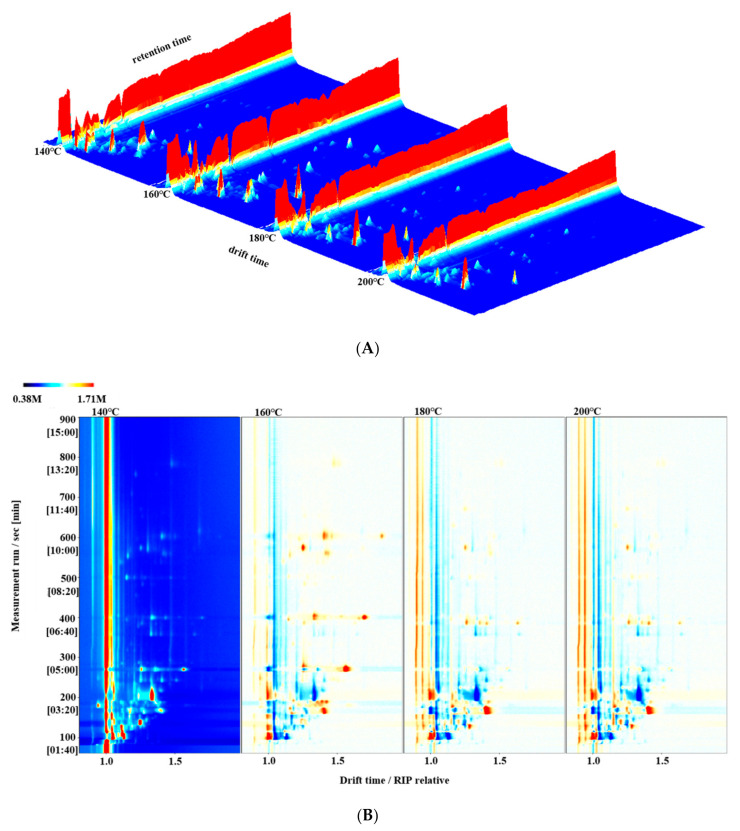
3D-topographic plots (**A**) and 2D-topographic plots (**B**) of volatile organic compounds in fish filets fried at different temperatures with a moisture content of 65%. Note: The *X*-axis represented the ion drift time of volatile organic compounds, the *Y*-axis represented the gas phase retention time, and the *Z*-axis represented the peak intensity.

Using the equation for fish filet moisture loss at different frying temperatures, shown in Figure 1, we could determine the time required to reach a water content of 65% at different frying temperatures (3 min 15 s, 2 min 52 s, 2 min 15 s, and 1 min 51 s for 140, 160, 180, and 200 °C, respectively). VOC changes under different frying conditions with a moisture content of 65% were analyzed using HS–GC–IMS. As shown in Figure 5A and B, the VOC types in the different samples were quite similar, but the signal intensity was slightly different. Figure 5B shows the VOC 2D topographic plots of fish filets under different frying conditions with a moisture content of 65%. Each point on the right side of the reactive ion peak indicated a volatile flavor compound or a dimer of volatile flavor compounds. The difference comparison model was applied to compare the differences of fish filets under different frying conditions with a moisture content of 65%. The samples without frying did not have fried aroma and attractive color (by sensory evaluation), and their shapes were easily destroyed. Therefore, the fried fish filets at 140 °C were used as a reference, and the topographic plot of other samples was subtracted from this reference. If the VOCs were identical, the background was white after deduction. However, red refers to the concentration of the substance, which was higher than the reference. In contrast, values lower than the reference are indicated by blue [11]. The signals of certain VOCs in fish filets declined or increased, or the signal intensity disappeared after processing. This could be attributed to the increase in temperature, and certain VOCs possibly affected by the temperature might have been easily degraded and decomposed. In addition, due to fat oxidation, the signals of some VOCs increased in a temperature-dependent manner.

### 3.6. Differences of Volatile Compounds in Different Frying Temperatures of Self-Heating Fried Fish Filets

**Figure 6:**
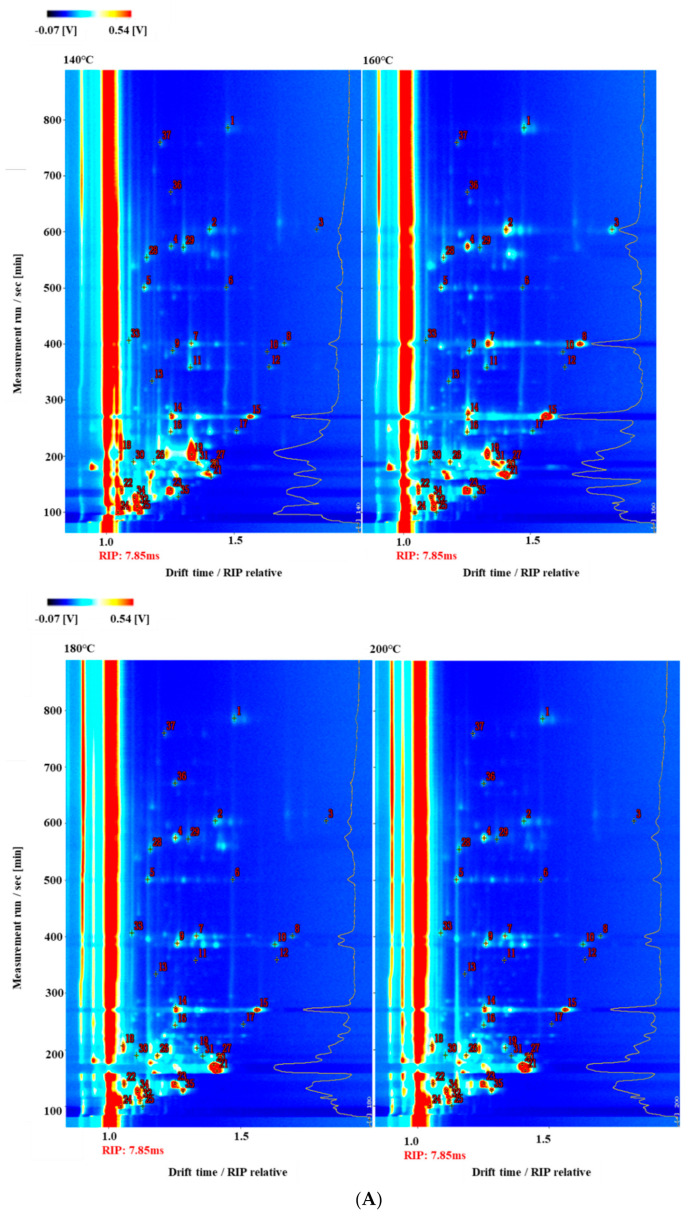

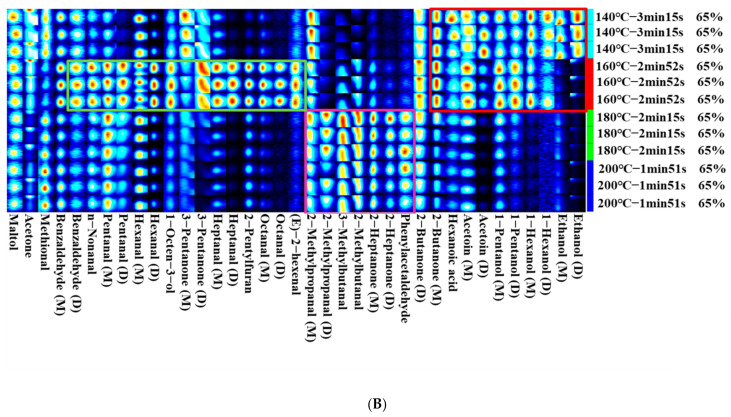
Topographic plots of GC–IMS spectra (**A**) with the selected markers obtained and fingerprint (**B**) comparison of VOCs with samples in different fried temperatures with a moisture content of 65%. Note: In the fingerprint, the darker the spot is, the larger is the quantity of volatile compounds. Each row represents all the signal peaks selected in a sample. Each column represents the signal peak of the same volatile compounds in different samples.

To distinguish between the changes in the VOCs in samples under different frying temperatures, 37 typical VOCs including 16 aldehydes, 9 alcohols, 9 ketones, 1 acid, 1 phenol, and 1 furan, from topographic plots were identified based on the drift and retention times (Figure 6A and Table 1). As shown in Figure 6A and Table 1, some VOCs were examined by both monomer and dimer forms such as octanal, benzaldehyde, heptanal, and 2-heptanone. The reason for this phenomenon was that the monomer and dimer forms of the same compound could exhibit similar retention times coupled with different migration times, correlating with the substance contents [31]. In the ionization region, a high level of content could accelerate the combination of neutral molecules and protonate molecules to form dimers, and a high proton affinity of analytes could also form dimers [32,33].

To clearly compare the differences between the VOCs among the fried samples at different temperatures and times with a moisture content of 65%, we set up the characteristic fingerprints corresponding to each temperature. As shown in Figure 6B, the gallery plots illustrate the differences in VOC concentrations from samples fried at different temperatures (140 °C, 160 °C, 180 °C, and 200 °C). Lipid and protein auto-oxidation could make samples form VOCs, which are highly related to the fried conditions. Additionally, the formation of some VOCs after frying could be a result of the Maillard reaction, which is a complicated reaction of forming aldehydes, ketones, pyrazines, furans, and other flavor compounds, and could be induced by certain factors (e.g., processing temperature and time, reaction chemistry, and system composition) [34,35]. In the red frame area, certain VOC contents (e.g., ethanol, 1-hexanol, and acetoin) visibly decreased simultaneously with increasing temperature. The signal intensities of 1-hexanol, 1-pentanol, acetoin, hexanoic acid, and 2-butaone from the samples fried at 140 °C and 160 °C were much stronger than those at 180 °C and 200 °C, suggesting that they might be volatilized at higher temperatures. This result might be influenced by the chemical volatilization and peroxidation of polyunsaturated fatty acids [36]. Alcohols, such as ethanol, 1-hexanol, and 1-pentanol, are often considered significant components of meat volatiles, as they are closely related to the typical fatty meat flavor. Zhang et al. [11] also found ethanol, 1-hexanol, and 1-pentanol in dry-cured fish and thought 1-pentanol might make an important contribution to the product aroma. The formation of acetoin might result from the production and processing environment [11]. However, in the purple frame area, the signal intensities of phenylacetaldehyde, 2-heptanone, 2-methylbutanal, and 3-methylbutanal were enhanced markedly after frying at 180 °C compared with the signal intensities obtained after frying at 140 °C and 160 °C. Lipid thermal degradation accelerated significantly with increasing temperature, which could contribute to the formation of certain aldehydes [37]. A previous study described 3-methylbutyral, generated by microbial degradation of amino acids or Strecker degradation of leucine, as a sort of deterioration in fish products [38]. In the green frame area, the VOC contents of the samples fried at 160 °C (e.g., (E)-2-hexenal, octanal, 2-pentylfuran, pentanal, 3-pentanone, 1-octen-3-ol, hexanal, heptanal, pentanal, n-nonanal, and benzaldehyde) were the highest compared with those observed at other frying temperatures. This might be due to the oxidative thermal degradation of polyunsaturated fatty acids at high frying temperatures or long frying times, producing many other volatile substances. Aldehydes, oily, and fruity aromas in foods are formed by the oxidative degradation of fatty acids, deamination, and degradation of amino acids, which can have a synergistic effect with other flavor substances, making a great contribution to the flavor profile of food due to their low olfaction thresholds. According to previous reports, five saturated linear aldehydes, including hexanal, heptanal, octanal, nonanal, and pentanal, have been discovered in many diverse aquatic products, and they have been deemed to be critical volatile flavor substances [39]. Also, 2-Pentylfuran, a non-carbonyl oxidation product, stemmed from linoleic and other n-6 PUFAs, plays a vital role in the overall aroma of meat products. A strong mushroom odor, 1-octen-3-ol, has usually been found in the flavor of fish products [40], primarily formed by the effect of 1,5-lipoxygenase on eicosapentaenoic acid (EPA, 20:5n-3) [41]. During frying, the Maillard reaction of the amino compounds and reducing sugars could give rise to the formation of sulfur compounds (e.g., methional) from methionine [42]. The methional content was the lowest in samples fried at 160 °C and increased notably at other frying temperatures. Due to volatilization, thermal degradation, and other chemical reactions, VOC damage was more serious with increasing temperatures or time, with the same moisture content. Therefore, we did not test high temperatures or long frying times for the retention of volatile aldehydes in fried fish filets.

### 3.7. Principal Component Analysis (PCA)-Based Fingerprint Similarity Analysis

**Figure 7:**
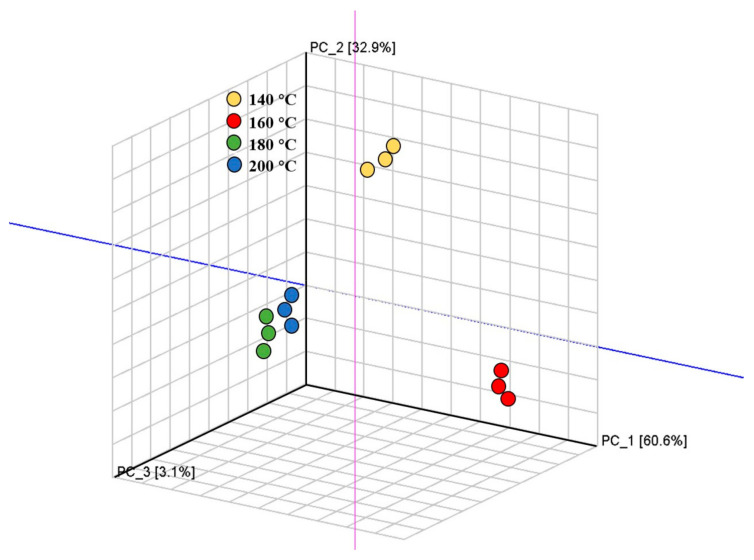
Three-dimensional principal component analysis (PCA) scores of volatile organic compounds (VOCs) in fish filets samples of different temperatures with a moisture content of 65%.

PCA was set up using signal intensities to highlight VOC dissimilarities. The higher the contribution rate, the better the principal components presented information about the original multi-index [43]. The PC scores of the spectroscopy data from GC-IMS at different temperatures are shown in Figure 7. The complex data of GC-IMS spectroscopy were reduced to several principal components for better presentation and analysis [44]. The total contribution of the first three components was 96.6%, among which PC1, PC2, and PC3 accounted for 60.6%, 32.9%, and 3.1%, respectively. The different colored areas represented four fish filets with different treatments, and the PCA results explicitly showed that samples fried at different temperatures under the same moisture condition could be separated and distinguished well in the distribution map. At frying temperatures of 140 °C, 160 °C, and 180 °C, the VOC samples varied greatly. The flavor of the samples fried at 180 °C and 200 °C was relatively similar. The data of the HS-GC-IMS analysis covered valuable information and could be a potentially useful tool to identify Spanish mackerel samples under different frying conditions using non-targeted characteristic markers.

## 4. Conclusions

In this study, we studied how different moisture contents and different frying temperature times with a constant moisture content could affect the texture, color, microstructure, and VOC characteristics of Spanish mackerel. The quality of the fried fish filet was optimal with a moisture content of 65%. Moreover, the content of certain VOCs from the samples fried at 160 °C (e.g., (E) -2-hexenal, octanal, 2-pentylfuran, pentanal, 3-pentanone, 1-octen-3-ol, hexanal, heptanal, pentanal, n-nonanal, and benzaldehyde) was the highest compared to samples fried at other temperatures, providing samples with a greasy or fried aroma. Based on the PCA, VOC samples varied greatly at frying temperatures of 140 °C, 160 °C, and 180 °C. The flavor of the samples fried at 180 °C and 200 °C was relatively similar. Based on the physicochemical properties and odor, it was found that the optimal condition for self-heating fried Spanish mackerel preparation was 160 °C for 2 min 52 s, which could result in good food quality and an appealing flavor to satisfy consumer preferences.

## Figures and Tables

**Table 1:** Volatile organic compounds from fish filets under different fried conditions with a moisture content of 65%.

Count	Compound	CAS#	Formula	MW	RI ^1^	Rt ^2^	Dt ^3^
1	n-Nonanal	124-19-6	C_9_H_18_O	142.2	1100.0	785.047	1.47752
2	Octanal (Monomer)	124-13-0	C_8_H_16_O	128.2	1010.7	604.389	1.40582
3	Octanal (Dimer)	124-13-0	C_8_H_16_O	128.2	1010.4	603.865	1.82482
4	2-Pentylfuran	3777-69-3	C_9_H_14_O	138.2	994.0	574.541	1.25607
5	Benzaldehyde (Monomer)	100-52-7	C_7_H_6_O	106.1	956.9	500.183	1.14933
6	Benzaldehyde (Dimer)	100-52-7	C_7_H_6_O	106.1	957.2	500.707	1.47114
7	Heptanal (Monomer)	111-71-7	C_7_H_14_O	114.2	897.3	400.569	1.33497
8	Heptanal (Dimer)	111-71-7	C_7_H_14_O	114.2	897.6	400.906	1.69924
9	2-Heptanone (Monomer)	110-43-0	C_7_H_14_O	114.2	888.4	387.755	1.26157
10	2-Heptanone (Dimer)	110-43-0	C_7_H_14_O	114.2	887.0	385.731	1.63128
11	1-Hexanol (Monomer)	111-27-3	C_6_H_14_O	102.2	865.7	357.743	1.33089
12	1-Hexanol (Dimer)	111-27-3	C_6_H_14_O	102.2	866.8	359.092	1.63944
13	(E)-2-hexenal	6728-26-3	C_6_H_10_O	98.1	845.6	333.127	1.18137
14	Hexanal (Monomer)	66-25-1	C_6_H_12_O	100.2	790.5	274.022	1.25582
15	Hexanal (Dimer)	66-25-1	C_6_H_12_O	100.2	786.8	270.378	1.56279
16	1-Pentanol (Monomer)	71-41-0	C_5_H_12_O	88.1	758.2	243.419	1.25456
17	1-Pentanol (Dimer)	71-41-0	C_5_H_12_O	88.1	759.8	244.876	1.51247
18	Acetoin (Monomer)	513-86-0	C_4_H_8_O_2_	88.1	713.8	206.623	1.05579
19	Acetoin (Dimer)	513-86-0	C_4_H_8_O_2_	88.1	708.5	202.615	1.33634
20	2-Methylbutanal	96-17-3	C_5_H_10_O	86.1	657.3	173.106	1.4005
21	3-Methylbutanal	590-86-3	C_5_H_10_O	86.1	633.9	162.541	1.41182
22	2-Butanone (Monomer)	78-93-3	C_4_H_8_O	72.1	577.1	139.589	1.06082
23	2-Butanone (Dimer)	78-93-3	C_4_H_8_O	72.1	578.1	139.953	1.25456
24	Ethanol (Monomer)	64-17-5	C_2_H_6_O	46.1	450.9	99.514	1.04846
25	Ethanol (Dimer)	64-17-5	C_2_H_6_O	46.1	453.6	100.242	1.13192
26	Pentanal (Monomer)	110-62-3	C_5_H_10_O	86.1	690.4	189.5	1.1863
27	Pentanal (Dimer)	110-62-3	C_5_H_10_O	86.1	690.4	189.5	1.42404
28	1-Octen-3-ol	3391-86-4	C_8_H_16_O	128.2	984.3	553.994	1.16002
29	Hexanoic acid	142-62-1	C_6_H_12_O_2_	116.2	993.1	572.601	1.30267
30	3-Pentanone (Monomer)	96-22-0	C_5_H_10_O	86.1	690.2	189.344	1.10834
31	3-Pentanone (Dimer)	96-22-0	C_5_H_10_O	86.1	688.7	188.305	1.35869
32	Acetone	67-64-1	C_3_H_6_O	58.1	488.2	109.994	1.1234
33	Methional	3268-49-3	C_4_H_8_OS	104.2	901.1	406.224	1.09037
34	2-Methylpropanal (Monomer)	78-84-2	C_4_H_8_O	72.1	540.9	126.679	1.11273
35	2-Methylpropanal (Dimer)	78-84-2	C_4_H_8_O	72.1	543.7	127.642	1.28368
36	Phenylacetaldehyde	122-78-1	C_8_H_8_O	120.2	1046.3	670.867	1.25446
37	Maltol	118-71-8	C_6_H_6_O_3_	126.1	1088.6	759.285	1.212

CAS is the registration number of chemical substances by Chemical Abstracts Service. ^1^ represents the retention time in the capillary GC column; ^2^ represents the retention index calculated on FS-SE-54-CB column using n-ketones C_4_–C_9_ as external standard; ^3^ represents the drift time in the drift tube.

## Data Availability

The data showed in this study are contained within the article.
